# Polyunsaturated Fatty Acids and Diabetic Kidney Disease: What have We Learned so Far?

**DOI:** 10.1007/s13668-026-00800-1

**Published:** 2026-09-17

**Authors:** Baris Afsar, Rengin Elsurer Afsar

**Affiliations:** 1 School of Medicine, Department of Nephrology, Saint Louis University, SSM Health Saint Louis University Hospital, St Louis, MO USA; 2 Department of Nephrology, Suleyman Demirel University Hospital, Cunur, Isparta, 32260 Turkey

**Keywords:** Diabetic kidney disease, Docosahexaenoic acid, Eicosapentaenoic acid, Fish oil, Omega 3 fatty acid

## Abstract

**Purpose of Review:**

Despite various renoprotective agents, diabetic kidney disease (DKD) still progresses in some patients necessitating further novel therapeutic options. With regard to diet and healthy eating patterns, one dietary intervention for DKD is polyunsaturated fatty acids (PUFAs) supplementation yet the full scope of these this intervention is incompletely understood. In this narrative review, we summarized the literature (both experimental and clinical studies) specifically investigating the relationship between omega fatty acids and DKD. Additionally, we explored the potential molecular effects of omega fatty acids in DKD, identify knowledge gaps and put proposals for future studies.

**Recent Findings:**

PUFAs supplementation decreased blood pressure, total cholesterol, low density lipoprotein cholesterol, triglyceride levels, proteinuria/albuminuria and kidney injury and inflammatory markers. Furthermore, structural alterations such as tubulointerstitial fibrosis, mesangial expansion, capillary and tubular dilatation, podocyte foot process effacement, glomerular basal membrane thickening are reduced by PUFAs although some studies showed neutral effects.

**Summary:**

PUFAs supplementation is a feasible option for DKD although studies are heterogeneous but no safety signals raised. More studies are needed to understand robust relationship between PUFAs supplementation and DKD.

## Introduction

In patients with diabetes mellitus (DM), diabetic kidney disease (DKD) occurs in up to 40% of patients. Despite various reno-protective agents, DKD still progresses in some patients necessitating further novel therapeutic options [1]. DKD is characterized by persistent albuminuria, a progressive decline in glomerular filtration rate (GFR), and associated with cardiovascular (CV) morbidity and mortality [2]. Managing DKD needs multitask approach including diet and healthy eating patterns [3], exercise [4], and medical therapy including renin-angiotensin-aldosterone system inhibitors (RASi), sodium-glucose cotransporter-2 inhibitors (SGLT2i), glucagon-like peptide-1 receptor agonists (GLP-1 RAs) and non-steroidal mineralocorticoid receptor antagonists (nsMRAs) [2]. With regard to diet and healthy eating patterns, protein restriction [5], plant based diet [6, 7]. Mediterranean diet [8, 9], dietary Approaches to Stop Hypertension (DASH) diet [9, 10] were all promising diet approaches in DKD.

Another potential dietary intervention for DKD is polyunsaturated fatty acids (PUFAs) supplementation containing omega 3 and omega 6 fatty acids.

Omega 3 and omega 6 PUFAs have an important role in the development growth, and normal function of tissues. The PUFA most prominently linked to physiological and biochemical actions are eico­ sapentaenoic acid (C20:5n-3, EPA) and docosahexaenoic acid (C22:6n-3, DHA) from the n-3 family, and arachidonic acid (C20:4n-6, AA) from the n-6 family. Humans can consume EPA and DHA directly from fish and seafood sources or synthesize these PUFA from their metabolic precursors, namely, the nutritionally essential α-linolenic acid (C18:3n-3, ALA) for n-3 PUFA and linoleic acid (C18:2n-6, LA) for n-6 process requiring desaturation and elongation re­ actions and a final peroxisomal β-oxidation step. Aging, smoking, insulin, glucagon, oxidative stress, zinc, magnesium, vitamin B6 may all effect these steps [11–13]. PUFA synthesis activity has been reported in the kidney with can be important in the context of DKD [12].

Studies have performed to investigate fish oil supplementation in patients with diabetes mellitus. A previous review suggested but do not firmly establish that long-chain omega-3 and omega-6 fatty acids reduce albuminuria in DKD [14]. A more recent meta-analysis showed that omega-3 fatty acids ameliorate proteinuria in patients with DKD. However, the beneficial effect is valid for patients who take omega 3 for at least 24 weeks suggesting heterogeneity among studies. Furthermore, this meta-analysis only included 344 participants [15]. However, some studies showed that fish oil supplementation has no effect on DKD [16–19] augmenting the divergence. In this narrative review, we extensively reviewed the literature (both experimental and clinical studies) specifically investigating the relationship between omega fatty acids and DKD. Additionally, we explore the potential molecular effects of omega fatty acids in DKD, identify knowledge gaps and put proposals for future studies.

## Existing Literature Regarding Polyunsaturated Fatty Acids and Diabetic Kidney Disease

The experimental and clinical studies specifically addressing the relationship between PUFAs and DKD are summarized in Tables 1 and 2 respectively. Experimental studies consistently showed that urinary protein/albumin excretion was decreased by PUFAs [20–26]. Additionally, experimental studies showed other beneficial effects of PUFAs such as decreased total cholesterol, low-density lipoprotein (LDL) cholesterol, very low-density lipoprotein (VLDL) cholesterol and triglycerides (TG) [20, 21], decreased kidney injury and inflammatory markers such as monocyte chemoattractant protein 1, transforming growth factor beta 1, type I collagen, sterol regulatory element-binding protein 1, intercellular adhesion molecule-1, fibronectin, neutrophil gelatinase-associated lipocalin (NGAL) and kidney injury molecule-1 (KIM-1) [21, 23, 24, 26]. Experimental studies also showed that structural pathological changes such as extracellular matrix accumulation tubulointerstitial fibrosis, mesangial expansion, capillary dilatation, tubular dilatation, podocyte foot process effacement, glomerular basal membrane thickening reduced by PUFAs [21–23, 27]. There is also reduced macrophage infiltration, phosphorylation of nuclear factor kappa-light-chain-enhancer of activated B cells (NF-κB) in kidneys [21–23], oxidative stress and apoptosis [25] With regard to glucose, some studies showed decreased levels [26], while most showed no effect on blood glucose levels [20, 21, 23, 25, 27, 28].

**Table 1:** Experimental studies specifically investigating the impact of polyunsaturated fatty acids in diabetic kidney disease

References	Methods	Results (Proteinuria/GFR)	Other Findings
Sinha et al. [20]	-Rats with DM fed with isocaloric diets with fish oil (20% w/w) or beef tallow (BT; 20% w/w) for 20 weeks -GFR was determined by inulin clearance	-DM rats exhibit glomerular hyperfiltration which was not affected by fish oil 24-hour UPE (mg/day) significantly decreased with fish oil (from 10.6 ± 1.8 to 4.6 ± 0.9, *P* < 0.01) but remained same in BT	-Weight gains, glucose BPs, insulin requirements and HDL**-cholesterol** did not change with Fish oil -Fish oil decreased VLDL-cholesterol, LDL **cholesterol** and **TG**
Barcelli et al. [28]	-Streptozotocin induced diabetic Sprague-Dawley rats divided into (I) Beef tallow diet (BT; rich in saturated fatty acids), (II)Evening primrose oil (rich in gammalinolenic and linolenic acids (III)Safflower oil (rich in linolenic acids) (IV) Fish oil (rich in EPA and DHA)	- Evening primrose oil and Safflower oil but not fish oil diet had beneficial effect on proteinuria	-Fish oil diet decreased plasma lipids and inhibited eicosanoid synthesis by platelets and kidney cortex.
Zhang et al. [21]	- KKAy/Ta diabetic mice received EPA ethyl ester at 1 g/kg per day or intraperitoneal saline injection for 8 weeks (control)	Baseline urinary ACR (mg/g Cr) were 386.0 ± 36.9 and 321.9 ± 51.2 in control and EPA treatment groups respectively (NS) -ACR at 4 weeks were 587.4 ± 31.6 and 161.3 ± 34.4 in control and EPA treatment groups respectively (P:0.0002) - ACR at 8 weeks were 610.4 ± 79.8 and 188.3 ± 34.6 in control and EPA treatment groups respectively (P:0.0019) -	-Systolic BP, fasting blood glucose, Total-C was not changed but Serum TG lowered by EPA -Tubulointerstitial fibrosis, extracellular matrix accumulation, glomerular macrophage infiltration decreased by EPA -Protein and gene expression levels of monocyte chemoattractant protein 1, transforming growth factor beta 1 and type I collagen, inhibited by EPA
Garman et al. [22]	- Sprague-Dawley rats randomly divided into: (I) nondiabetic (ND) (II) streptozotocin-induced diabetic (D) (III) D + n-3 PUFA diet (D+canola), (IV) D + n-6 (omega-6) PUFA diet (D+corn) for 30 weeks **-**CrCl was calculated based on plasma and urine creatinine concentrations and 24-h urine output	-D+canola and D+corn reduced the diabetes associated increase in urinary UAE by 11.7- and 2.9-fold, respectively -UAE (Mg/day) was higher in D rats vs. ND rats 97.3 ± 9.4 vs. 17.8 ± 6.4) -UAE significantly ameliorated by n-3 PUFA diet (D+canola): 8.3 ± 2.2 mg/day) and D+corn), 33.8 ± 6.1 mg/day) respectively -CrCl was 2.2 + 0.1, 1.8 + 0.3, 2.1 + 0.5, and 1.8 + 0.3 (ml/min) for the ND, D, D+canola, and D+corn animals (NS)	-The D1corn animals showed a 3.9-fold increase in blood glucose, and 0.80-fold decrease in body weight compared with non-diabetic diet -Mesangial expansion, capillary dilatation, tubular dilatation, and accumulation of inflammatory cells partially attenuated in the D+corn group.
Chin et al. [23]	-db/db mice received Omacor (composed of DHA and EPA (0.2 g/100/day) or olive oil (0.2 g/100) g/day) for 2 weeks	-Albuminuria similar between groups at baseline -After 2 weeks, albuminuria was lower in the Omacor group vs. olive oil group (242.0 ± 148.0 vs. 356.1 ± 128.1 µg/day/100 g (P:0.002) -Serum creatinine (0.57 ± 0.02 vs. 0.75 ± 0.02 mg/dL, P:0.001) and CrCl (60.3 ± 14.4 vs. 125.4 ± 19.0 µl/min/100 g body weight P:0.035) were lower in the Omacor group vs. olive oil group at 2 weeks	-In kidney tissue Omacor decreased -TG content -Sterol Regulatory Element-Binding Protein 1 -Expansion of mesangial matrix -Expression of desmin phosphorylation of NF-κB
Taneda et al. [25]	- Non-diabetic CD1 mice and diabetic CD1 mice (by i.p injection of STZ) divided into (I) Nondiabetic mice with standard chow (II)Non-diabetic mice 5% EPA (wt/wt) (III): standard chow (IV) standard chow + 5% (EPA) (V) standard chow and injected with insulin for 2 weeks -Tubular cells (NRK-52E) used for in vitro experiments	-In non dıabetic mice with and without 5% EPA, UAE (µg)/Cr (mg) were 50.4 ± 26.3 and 48.7 ± 17.1 respectively - UAE increased in DM rats with chow diet to 625.4 ± 336.1 but decreased significantly by 5% EPA (150.8 ± 93.8) and insulin respectively (96.3 ± 35.1)	-In vitro 5% EPA suppressed oxidative stress and mitochondrial apoptosis
Kim et al. [24]	-4 groups of rats: (I) Control, (II) Diabetic, (III)Control+gamma linolenic acid (GLA) (IV) Diabetic + GLA for 3 months - GLA dose was 40 mg/kg/day	-24-hr UAE (mg/day) was 0.31 ± 0.09 in rats in group III. - Compared to the C group (0.35 ± 0.07mg/day), 24-hour urinary albumin excretion at three months was significantly higher in DM rats (2.51 ± 0.28 mg/day) (*p* < 0.01), and GLA treatment significantly reduced albuminuria in DM rats (1.11 ± 0.12 mg/day) (*p* < 0.05)	- In kidneys, intercellular adhesion molecule-1, monocyte chemoattractant protein-1, and fibronectin mRNA and protein expression levels were significantly higher in DM than in control group but significantly abrogated by GLA
El-Boshy et al. [26]	- STZ induced DM in Wistar rats divided into (I) No ıntervention (II) Paricalcitol (Pcal) as 0.25 mg/kg/day 5 times/week) (III) Oral omega 3 as 415 mg/kg/day 5 times/week (IV) Pcal+ oral omega 3 For 8 weeks **-**CrCl calculated as: [Urine creatinine (mg/dl) xUrine flow (ml/min)]/ Serum creatinine (mg/dl)	- CrCl (mL/min) were 0.28 ± 0.03, 0.36 ± 0.06, 0.37 ± 0.08 and 0.51 ± 0.08 group 1,2,3 and 4 respectively (*P* < 0.01) -24 h UPE increased in group 1 but significantly decreased in group 3 and group 4 (absolute data not given)	-Hyperglycemia, dyslipidemia, apoptosis NGAL/KIM-1 and increased caspase-3 expression, occurred in DM rats -These alterations are ameliorated with omega 3 alone and more with Pcal+omega 3 combination
Lee et al. [27]	-Mice experimentally divided into (I) db/m (Control) (II) db/db mice fed with soybean oil (III): db/db mice fed with olive oil (V): db/db mice fed with EPA At a dose of 250 µM 5%	-No data of proteinuria/albuminuria and GFR	- Wilms tumor protein-1 (WT1) expression in podocytes significantly decreased in db/db mice Show Significant Increase in -Mean Glomerular Area, glomerular basal membrane width, Podocyte Foot Process Effacement increased in db/db mice vs. db/m mice - Olive Oil or Fish Oil improved WT1 Expression and reduced glomerular enlargement, glomerular basal membrane thickening, and podocyte foot process effacement

DM: Diabetes Mellitus, GFR: Glomerular Filtration rate, UPE: Urinary Protein Excretion: BP: Blood Pressure, HDL-C: High-density lipoprotein cholesterol, VLDL-C: Very-low-density lipoprotein cholesterol, LDL-C: Low-density lipoprotein cholesterol, TG: Triglyceride, EPA: Eicosapentaenoic acid DHA: docosahexaenoic acid, ACR: Albumin to creatinine ratio,, Total-C: Total Cholesterol, PUFA: polyunsaturated fat, CrCl: Creatinine clearance, NF-κB: nuclear factor kappa-light-chain-enhancer of activated B cells STZ: Streptozotocin, NGAL: neutrophil gelatinase-associated lipocalin, KIM-1: Kidney injury molecule 1

**Table 2:** Clinical studies specifically investigating the impact of polyunsaturated fatty acid in diabetic kidney disease

References	Patient/Methods	Proteinuria/GFR)	Other Results
Haines et al. [16]	-41 patients with T1DM randomized to: -Fish oil 15 g (treatment) (*N* = 19) vs. olive oil (placebo) (*N* = 22) for 6 weeks -The fish oil was provided 2.7 gm EPA and 1.9 gm DHA daily	No change in albuminuria (absolute data not given)	-LDL-C increased in treatment group vs. Placebo -Significant reduction in thromboxane production by platelets with fish oil
Jensen et al. [33]	− 18 patients with T1DM randomized to: − 4.6 g EPA + DHA daily (21 g cod-liver oil daily) vs. 21 g olive oil daily (control) for 8 weeks	-No change in 24-UAE and GFR in both groups after 8 weeks	-Systolic BP decreased in intervention group but not in olive oil group -Glucose, HbA1c, insulin dose did not change in both groups -Transcapillary escape of albumin decreased in intervention group but not in olive oil group independently of BP reduction
Hamazaki [29]	-EPA ethyl ester (1.8 g/d) was administered to 5 insulin dependent and 11 noninsulin-dependent patients with diabetes for 6 months	-Geometric mean of spot urine albumin excretion (mg/g) significantly reduced from 65 to 36 (p<:0.001) with EPA -Serum creatinine, blood urea nitrogen did not change with EPA	-No change in body weight, BP, Fasting blood **glucose**, HbA1c, Total-C and TG
Shimizu et al. [30]	-45 patients with T2DM - EPA (900 mg/day (*N* = 29) and non EPA-treated group(*N* = 16) (controls) for 12 months -UAE determined by urine albumin index (UAI)	-Baseline UAI (mg/gCr) were 200.1 ± 60.0 in control group and 447.2 ± 126.3 in EPA treated group -At 3 months UAI were 108.2 ± 14 and 57.8 ± 6.7 in control vs. EPA treated group (*P* < 0.01) -At 12 months UAI were 114.4 ± 12 and 59.2 ± 11.3 in control vs. EPA treated group (*P* < 0.01)	-EPA did not affect BPs, fasting glucose, **HbA1c**, Total-C, HDL-C and TG
Lungershausen et al. [17]	-Patients with T1DM and T2DM -Baseline UAE: 20–200 pg/min (30–300 mg/24 h) -Randomization to Fish oil containing 2 g EPA and 1.4 g DHA (*N* = 16) vs. 16 corn oil (*N* = 16) (placebo) for 12 weeks	There was no treatment effect on albumin excretion -UAE at baseline and 12 weeks in fish oil group were 60 to 70 µg/min respectively (*P* > 0.05) -UAE at baseline and 12 weeks in corn oil group were 83 to 95 µg/min respectively (*P* > 0.05) -No change in CrCl in both groups	-Systolic BP decreased more in fish oil group vs. corn oil group (-5.9 ± 2.4 mmHg vs. -0.4 ± 1.5 mmHg **(P:0.039)** -No difference in diastolic BP -Body weight, fasting blood glucose HbA1c Total-C, HDL-C, LDL-C did not change between groups -Plasma TG decreased significantly with fish oil vs. corn oil
Rossing et al. [18]	− 31 patients with T2DM randomized to: -Fish oil (4.6 g omega3 fatty acids/day) (*N* = 14) vs. olive oil (*N* = 15) for 12 months -Albuminuria, GFR, (5lCr-labeled EDTA plasma clearance), 24-h ambulatory BP and lipid profile were determined every 6 months.	-Mean albuminuria increased by 22% (1–46%) in the fish oil group vs. 15% (11–49%) in the olive oil group (NS between groups). -Fractional albumin clearance was increased 33% the fish oil group and 23% in the olive oil group (NS between groups) - GFR decreased from 116 ± 7 to 105 ± 7 ml/ min/ 1.73 m^2^ (mean ± SE) vs. from 108 ± 6 to 103 ± 7, fish oil and olive oil, respectively (NS between groups).	-No significant changes occurred in 24-h ambulatory BP and HbA1c -Serum TG decreased from 0.97 (0.5-4.0) mmol/L to 0.8 (0.4-3.0) in fish oil group but increased from 1.01 (0.4-2.0) to1.09 (0.4-2.0) in the olive oil group (*P* < 0.05 between groups) -The decrease in VLDL-C was higher in fish oil group from 0.45 to 0.37 in fish oil group vs. olive oil group from 0.44 to 0.41 (*P* < 0.05)
Möllsten et al. [36]	- Nested case control study including patients with T1DM -75 patients with micro or macroalbuminuria, 225 patients with normoalbuminuria -Semi quantitative FFQ used for assessment of nutritional intake during the previous 12 months	-High fish protein consumption (≥ 75th percentile, intake 9.35 g fish protein/day, i.e.,∼53 g fish/day) associated with lower risk of microalbuminuria vs. low fish protein consumers (mean 2.72 g/day i.e., ∼15 g fish/day) (OR: 0.22, 95% CI: 0.09–0.56) -The same trend also observed for high intake of fish fat (OR: 0.31, 95% CI: 0.13–0.76)	-Total protein, meat protein, milk protein and vegetable protein did not differ between patients with micro and macroalbuminuria vs. normoalbuminuria
Lee et al. [37]	-Cross-sectional study included 517 patients with DM and 21,867 without DM (DM diagnosis by self-report) -Fish consumption categorized as less than 1, 1 to 2, and more than 2 portions/wk. by semi quantitative FFQ	- Fish consumption was associated with a lower risk of macroalbuminuria in participants with diabetes (OR: 0.22, > 2 versus < 1 portion/wk; 95% CI: 0.07–0.70; P:0.009) -No association observed between fish consumption and microalbuminuria in participants with and without DM	-Baseline total energy intake and fat intake is higher in high fish consumption with portions > 2 vs. portions < 1
Lee et al. [38]	-Observational cohort study -Individuals with T1DM participated in the Diabetes Control and Complications Trial between 1983 and 1993 included -Baseline dietary omega-3 PUFAs in g/day obtained from a modified Burke-type diet history -UAER was measured annually in a 4-h timed urine specimen. -Incident albuminuria (the first occurrence of UAER of > 40 mg/24 h sustained for ≥ 1 Year) recorded in normoalbuminuric individuals at baseline	-Among the 1,362 normoalbuminuric participants at baseline, 95 people developed albuminuria (mean follow-up of 6.5 years) -No association was found with incident albuminuria and omega 3 PUFAs in whole group -Higher intake of omega 3 PUFAs was associated with lower levels of UAER only in participants with values of A1C above the median (7.7%)	
Wong et al. [39]	-Double-blind placebo-controlled trial -97 T2DM patients without prior cardiovascular disease randomized to -Fish-oil (4 g⁄day, n:49) vs. olive-oil (with equivalent calories, as placebo, n:48) for 12 weeks	-Serum creatinine was lower (-4.5 µmol⁄l, P:0.01) in fish-oil-group vs. placebo -No data for proteinuria/albuminuria	-BPs, fasting blood glucose, Total-C, LDL-C, HDL-C, HbA1c, oxidative stress markers (isoprostane, superoxide dismutase) and hsCRP were not different between groups - Serum TG level lower in fish oil vs. placebo -No significant difference in Flow-mediated dilatation between groups
Miller et al. [34]	-Randomized, placebo-controlled study including patients with T2DM -2 period crossover trial of 4 g/day of omega 3 PUFA supplementation -Each period lasted 6 weeks and was separated by a 2-week washout − 29 patients finished both periods	- Omega 3 PUFA had no significant effects on 24-h UAE vs. placebo (-7.2%; 95% CI -20.6 to 8.5; *P* = 0.35 -In subgroup analyses, there were significant decreases in 24-h UAE using RASi (-17%) -No effect of PUFA on eGFR	-n-3 PUFA did not change SBP, DBP but significantly decrease TG -when compared with placebo, -Omega 3 PUFA supplementation increased glucose and alanine aminotransaminase -NGAL Neutrophil gelatinase-associated lipocalin decreased more with n-3 PUFA vs. placebo -No significant effect of Omega 3 PUFA on KIM-1 kidney injury molecule-1, N-acetyl β-d-glucosaminidase, liver fatty acid-binding protein cystatin C, and β2-microglobulin
Lee et al. [35]	-Randomized, double-blind, placebo-controlled study -19 patients with DN randomized to: Omega-3 FAs (Omacor 3 g/day) (*N* = 11) vs. olive oil 3 g/day for 12 weeks -One gram of Omacor contained 460 mg of EPA and 380 mg DHA	24-hour UPE in baseline and after 12 weeks in Omega-3 FA group were 0.60 ± 0.63 vs. 0.69 ± 0.65 (*P* > 0.05) -24-hour UPE in baseline and after 12 weeks in Olive oil group were 0.46 ± 0.44 ± 0.63 vs. 0.47 ± 0.41 (*P* > 0.05) -Estimated GFR (mL/min/1.73 m^2^) in baseline and after 12 weeks in Omega-3 FA group were 55.0 ± 8.0 vs. 59.2 ± 10.8 (*P* > 0.05) -Estimated GFR (mL/min/1.73 m2) in baseline and after 12 weeks in Olive oil group were 62.7 ± 7.4 vs. 63.4 ± 8.4 (*P* > 0.05)	-SBP, DBP, glucose, HbA1c, Total Chol, HDL-C, LDL-C, TG, NGAL levels similar after 12weeks between 2 groups - The erythrocyte membrane contents of omega-3 FA and EPA were significantly increased and omega-6/omega-3 FA ratio were significantly decreased after 12 weeks compared with the baseline values in the omega-3 FA group.
Han et al. [31]	-Retrospective study included 344 patients with T2DM with omega-3 fatty acid supplementation for hypertriglyceridemia -Change in Urine ACR and GFR assessed before and after omega-3 fatty acid supplementation	-Urine ACR was significantly decreased after omega 3 fatty acid supplementation (from 475.8 ± 1235.9 mg/g to 385.6 ± 1067.9 mg/g, p :0.003 - GFR change before and after supplementation (mL/min/1.73 m2) were 76.1 ± 25.9 vs. 74.4 ± 27.9, difference:-1.8 ± 11.2, P: 0.004	-Total-C cholesterol, fasting blood glucose, TG, hsCRP significantly reduced after omega 3 fatty acid supplementation -No change observed in HbA1c and HDL-Cholesterol
Elajami et al. [40]	-Patients with coronary artery disease with (*N* = 79) and without T2DM (*N* = 183) were randomized to Lovaza (*N* = 134) (1.86 g of EPA and 1.5 g of DHA daily) vs. no Lovaza (*N* = 128) for 1 year	- In subjects without DM, no change in urine ACR observed in either the Lovaza or control groups. -ACR increased 72.3% (*P* < 0.001) in subjects with DM subjects not receiving Lovaza -Subjects with DM receiving Lovaza had no change in ACR. -In subjects with diabetes on ACEi or ARBs urine ACR did not change with Lovaza but increased 64.2% in patients not receiving Lovaza (*P* < 0.001). -eGFR not changed in both groups with and without Lovaza	- Change in ACR was directly correlated with change in systolic BP -In patients with type 2 DM, Lovaza decreased TG levels but did not affect HbA1c, Glucose, SBP, DBP, Total-C, HDL-C and LDL-C
de Boer et al. [19]	-Randomized clinical trial with a 2 × 2 factorial design conducted among 1312 adults with T2DM -Participants were randomized to 4 groups: (I): vitamin D3 (2000 IU/d) + omega-3 fatty acids (EPA + DHA 1 g/d) (*N* = 370) (II): Vitamin D3 + placebo (N: 333), (III): Omega-3 fatty acids+placebo (*N* = 289), (IV): or 2 placebos (*N* = 320) for 5 years − 934 (71%) completed the study.	- There were no significant difference in change in eGFR at 5 years between groups - UAE also showed no statistically significant differences between groups	
Lin et al. [41]	− 169 patients with T2DM without DN and 144 patients with T2DM with DN (UACR of ≥ 30 mg/g) included -A semi quantitative food frequency questionnaire was administered	-Patients with DN had a significantly lower intake of high-fat marine fish, shellfish vs. patients with no DN -High-fat marine fishes (OR: 0.868; 95% CI: 0.781–0.965, p:0.009). and Shellfish OR: 0.709; 95% CI: 0.554–0.908 p:0.007) intake related with lower DN - Omega 3 PUFAs especially EPA (OR: 0.821; 95% CI: 0.688–0.979, p:0.029) and DHA (OR: 0.903; 95% CI: 0.823–0.992, *p* = 0.033)reduced DN risk, while omega-6 PUFAs did not	
Liu et al. [42]	-Cross-sectional study -444 healthy controls, 115 T2DM patients without DN 52 T2DM with DN patients included -Serum omega-3 PUFA measured by Liquid chromatography-mass spectrometry (LCMS)	-Serum omega-3 PUFA was significantly negatively correlated with albumin/creatinine ratio	
Elbarbary et al. [32]	-RCT including patients with T1DM and DN randomized to: -Omega-3 fatty acids capsules (1 g daily) (*N* = 35) or placebo (*N* = 35) for 6 months -Each capsule contains 1000 mg Fish Oil (180 EPA and 120 DHA per soft gel) -Nutrient intake was tabulated from 24-h dietary recall	- UACR (mg/g creatinine) decreased from 65.2 (45.6–125) to 31.5 (29-66.3) (*P* < 0.001) in omega 3 group but increased from 67.4 (43.2–108) to 88.1 (60.2-130.1) (*P* < 0.001) in placebo. group difference *P* < 0.001) - eGFR (ml/min/1.73 m^2^) baseline vs. 6 months 109.9 ± 6.74 vs. 113.7 ± 9.96 (P:0.096) in omega 3 group. -eGFR (ml/min/1.73 m2) baseline vs. 6 months 110.2 ± 7.47 vs. 108.3 ± 5.77 in placebo (P:0.074) (group difference for eGFR P:0.006)	-Omega-3 fatty acids decreased SBP, TG Total-C, LDL-C and increased HDL-C vs. baseline and placebo -Omega-3 fatty acids decreased KIM-1 vs. baseline and placebo
Nakamura et al. [43]	-Prospective cohort study including patients with T2DM and DN -Fatty acid intakes of EPA, DHA, arachidonic acid **linolenic** acid, gamma-linolenic acid, alpha linolenic acid calculated by dietary survey -The relationship between Fatty acid intakes and UAE investigated	− 28 patients had remission of DN (UAE < 30 mg/gCr) and DN progressed in 18 (UAE > 300 mg/gCr) after 36months of follow-up. -The ratios of omega-6/omega-3 fatty acids tended to be higher in the remission group vs. progression group but not significant	
Xu et al. [44]	-Patients with T2DM with (*N* = 135) and without DN (*N* = 322) were enrolled -Clinical data, serum levels of unsaturated fatty acids, and other laboratory indicators were collected.	-After adjusting for confounders, EPA (OR: 0.583, CI: 0.374, 0.908), DHA (OR: 0.826, CI: 0.716, 0.954), and n-3 DPA (OR: 0.513, CI:0.298, 0.883) were negatively associated with DN -Combination of EPA and DHA exhibited a significant inverse association with DN (OR:0.678 95% CI: 0.493–0.933)	-Combination of DHA and EPA decreased extracellular matrix production induced by transforming growth factor beta 1 in podocytes and tubular cells
Tian et al. [45]	-Large-scale prospective cohort study (The UK Biobank) -20,338 individuals with T2DM included. -Among them 5928 were Fish oil users and (*n* = 14 410) were Fish oil nonusers - At the baseline assessment center visit (2006–2010), participants demographic and socioeconomic factors, lifestyle factors (including the assessment of fish oil supplementation obtained	-During 13.2 years of follow-up, DKD developed in 2777 cases in 254 691person years -Use of fish oil is independently associated with lower incidence for development of DKD (HR: 0.87, 95% CIs: 0.79–0.95	-Fish oil use was associated with lower composite macrovascular complications, coronary heart disease, peripheral artery disease and diabetic retinopathy

T1DM: Type 1 Diabetes Mellitus, EPA: Eicosapentaenoic acid DHA: PUFA: Polyunsaturated fatty acid docosahexaenoic acid, LDL-C: Low-density lipoprotein cholesterol, UAE: Urinary Albumin Excretion, GFR: Glomerular Filtration Rate, BP: Blood Pressure, Total-C: Total Cholesterol, TG: Triglyceride, HDL-C: High-density lipoprotein cholesterol, CrCl: Creatinine clearance, VLDL-C: Low-density lipoprotein cholesterol Very Low-density lipoprotein cholesterol, FFQ: Food frequency questionnaire, LC-PUFAs: Long-Chain Poly unsaturated Fats, eGFR: Estimated Glomerular Filtration Rate, DN: Diabetic nephropathy, UACR: Urinary albumin to creatinine ratio, DPA: Docosapentaenoic acid, DKD: Diabetic kidney disease, NGAL: Neutrophil gelatinase-associated lipocalin, KIM-1: kidney injury molecule-1

The results of clinical studies are more heterogeneous compared to experimental studies. Urinary protein/albumin excretion was decreased by PUFAs [29–32], while other studies showed no effect [16–19, 33–35]. The impact of PUFAs on BPs, lipid levels, glucose levels and HbA1c were heterogeneous as either beneficial or neutral. Only one study showed that Omega 3 PUFA supplementation increased glucose and alanine aminotransaminase [34] and no other clinical studies showed harmful effects on albuminuria and other clinical and laboratory parameters (Table 2).

## Discussion

In this narrative review, we analyzed the existing literature about the relationship between DKD and PUFAs. The findings of our narrative can be summarized as (i): Animal studies consistently showed PUFAs are beneficial with regard to proteinuria/albuminuria (ii): Clinical studies are more heterogeneous and some showed beneficial and others showed neutral effects on proteinuria/albuminuria (iii): Both animal and clinical studies showed various favorable effects including metabolic, anti-inflammatory, anti-oxidant and structural impacts.

Although not homogeneous, the results of some of these studies are promising and points an important issue since high prevalence of diabetes is closely related to unhealthy diet, sedentary lifestyle, and obesity.

This supports the importance of intervention with PUFAs and to elaborate and understand the potential impacts of PUFAs in the context of DKD.

**Fig. 1:**
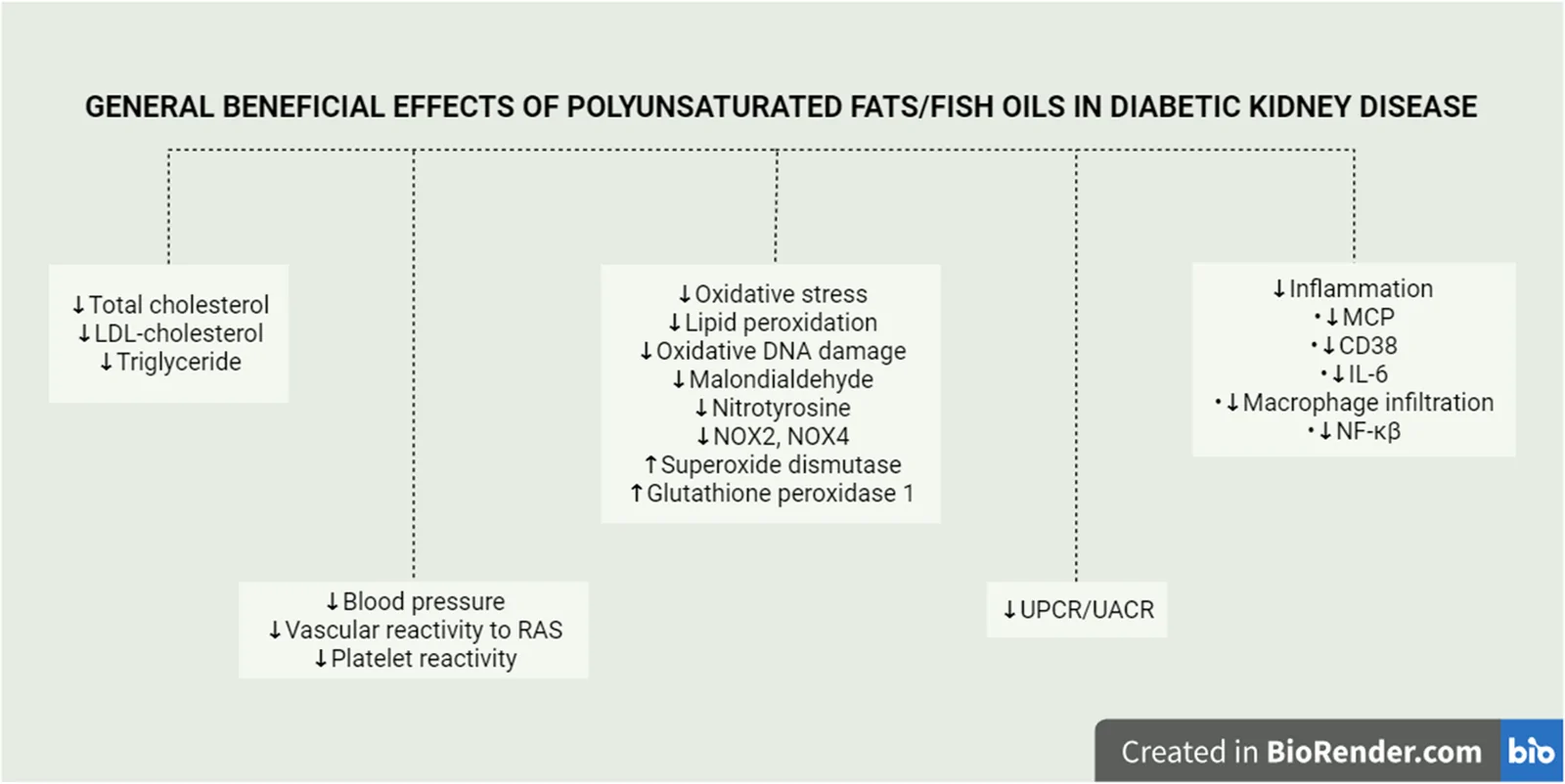
Favorable effects of polyunsaturated fatty acid/fish oil supplementation in hemodynamic, metabolic and inflammatory parameters. *Abbreviations*: PUFA: Polyunsaturated Fatty acid, RAS: Renin-Angiotensin System, NOX: NADPH oxidases, UPCR: Urinary protein/creatinine ratio, UACR: Urinary protein/creatinine ratio, MCP: Monocyte Chemoattractant Protein, IL-6: Interleukin 6, NF-κB: enhancer of activated B cells

**Fig. 2:**
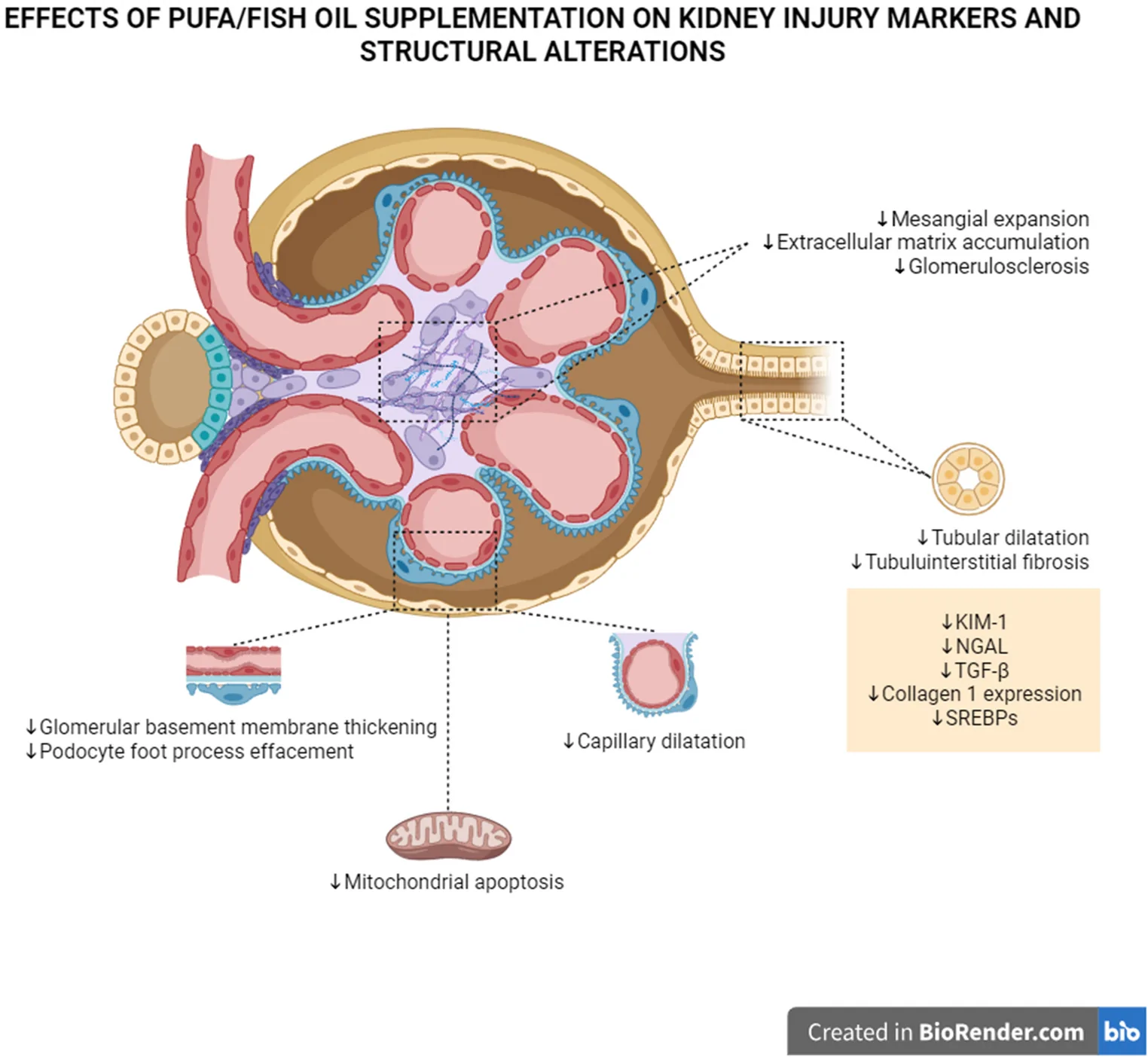
Effects of polyunsaturated fatty acid/fish oil supplementation on kidney injury markers and structural alterations. *Abbreviations*: PUFA: Polyunsaturated Fatty acid, KIM-1: kidney injury molecule-1, NGAL: Neutrophil gelatinase-associated lipocalin, TGF-β, Transforming growth factor beta, SREB1: Sterol Regulatory Element-Binding Protein 1

The pathogenesis of DKD is very complex. Pathologic alterations include formation of advanced glycation end-products (AGEs), activation of polyol, protein kinase C (PKC) and the hexosamine biosynthetic pathways, oxidative stress, endothelial dysfunction, mitochondrial dysfunction, lipid accumulation in podocytes and tubular cells, abnormal podocyte expression of vascular endothelial growth factor (VEGF), and podocyte/endothelial cell injury are all implicated [46, 47]. Since multiple pathways are activated, it is not expected that one single measure or medication will totally ameliorate or reverse the pathology in DKD. For this reason, combination of measures (lifestyle, exercise, drug combinations) are important for management of DKD. Indeed, now combination of drugs treatment (RAS blockers, SGLTi, GLP1RAs and nsMRAs) are now recommended in patient with progressive DKD [48]. Despite these advances, DKD still progress in some patients and new, feasible alternatives are needed. In this context, PUFA supplementation may be one of the auxiliary options in DKD. Indeed, some studies showed that PUFAs supplementation may decrease urinary protein/albumin excretion in DKD which is the hallmark of the disease. PUFAs supplementation has general beneficial effects (Fig. 1), and also specific favorable actions in kidney (Fig. 2).

In the next section, we will discuss the underlying mechanisms explaining beneficial effects of PUFAs in DKD.

### PUFAs Platelet Function Blood Pressure and DKD

PUFAs supplementation is reported to reduce BP and vascular reactivity. In an earlier study Lorenz et al. showed that omega-3 PUFAs [11% of EPA and 16% of docosahexaenoic acid (DHA)] decreased BP and vascular reactivity to angiotensin II and norepinephrine along with decreased platelet reactivity [49]. It was also shown that Omega-3 fatty Acids (500 g of mackerel per week for 4 weeks equivalent to approximately 1 g per day of EPA + DHA) also decreased platelet-monocyte aggregation [50]. Eicosapentaenoic acid also increase nitric oxide along with decrease thromboxane A2 production, platelet aggregation and vasoconstriction [51]. In a previous meta-analysis including 11 trials that enrolled normotensive individuals (n:728), omega-3 PUFA supplementation led to significant reductions of systolic BP and diastolic BP [52]. A more recent umbrella meta-analysis (The pooled estimate of 10 meta-analyses) indicated that n-3 PUFAs supplementation might play a role in improving SBP and DBP [53]. In the context of DKD some studies showed fish oil decreased SBP [17, 32, 33] while other showed neutral effect [18, 20, 21, 29, 30, 34, 39].

### PUFAs, Cholesterol and Glycemic Control in DKD

Abnormal fat metabolism is one of the benchmarks in DKD. These abnormalities can be summarized as: (i): abnormal uptake and oxidation of fatty acids by tubular cells causing lipid accumulation (ii): increased expression of sterol regulatory element-binding proteins (SREBP) and isoforms associated with cholesterol synthesis that mediate intracellular cholesterol sensing leads to renal damage in DKD. Inhibition of cholesterol efflux and increased cholesterol influx in DKD cells increases free cholesterol levels, which activate sterol O-acyltransferase 1 to form cholesteryl esters that are stored and cause excessive accumulation of lipids in podocytes (iii): Abnormalities in sphingolipid and phospholipid metabolism (iv): Abnormalities in lipophagy [54]. Furthermore, high serum TG levels were associated with prevalence of DKD and albuminuria in patients with DM [55–57]. It was also shown that high cholesterol levels [58] and high TG levels [57, 59] was associated with development of albuminuria.

In clinical trials in populations with diabetes, PUFAs supplementation decreased plasma TG levels and increased HDL-cholesterol levels [60, 61], and tissue accumulation of lipids and lipotoxicity in the kidney may underlie the development of diabetic nephropathy [62–65]. Specifically, in the context of DKD, experimental and clinical studies showed other beneficial effects of fish oil such as decreased Total cholesterol, LDL cholesterol, VLDL cholesterol and TG (Tables 1 and 2) [18, 20, 21, 31, 32]. However, Haines et al. showed that LDL cholesterol increased with fish oil [16].With regard to fasting blood glucose and HbA1c levels PUFAs supplementation has neutral effects (Tables 1 and 2).

### PUFAs, Inflammation, Oxidative Stress and DKD

The role of inflammation and oxidative stress in DKD is increasingly recognized. Increased activity of tumor necrosis factor-alpha (TNF-α), signaling and enhanced release of monocyte chemoattractant protein 1 are observed in DKD [66–68]. It was shown that interleukin-6 (IL-6) levels independently associated with an increased risk for progression of DKD [69]. Recently, it was suggested that instead of being innocent bystander, complement activation plays a role in in DKD [70, 71]. In DKD, PUFA supplementation decreased monocyte chemoattractant protein-1 [21, 22, 24], CD38 cells and IL-6 [22].

Oxidative stress in is another important factor for playing a role in DKD [72]. In DKD, reactive oxygen species are produced by include NADPH oxidases (Nox), uncoupled endothelial nitric oxide synthase (eNOS), xanthine oxidase (XO), cytochrome P450 (CYP450), and lipoxygenase and mitochondria. Oxidative stress triggers phosphoinositide 3-kinase/protein kinase B (PI3K/Akt), transforming growth factor beta/p38-mitogen-activated protein kinase (TGF-β/p38-MAPK), NF-κB, and the Janus kinase/signal transducer and activator of transcription (JAK/STAT) pathways leading to tissue damage [73]. Taneda et al. showed that in streptozotocin-induced diabetic mice, dietary eicosapentaenoic acid (EPA) reduced the levels of lipid peroxidation product (4-hydroxy-2-nonenal), oxidative DNA damage (8-hydoxy-deoxyguanosine) [25]. In diabetic mice, EPA decreased oxidative stress markers malondialdehyde (MDA) and nitrotyrosine [21]. In diabetic mice, EPA reduced Nox2, and Nox4 in the renal cortex [74]. Fish oil supplementation also increased anti-oxidant defense by upregulation of expressions of renal superoxide dismutase-1 (SOD-1) and glutathione peroxidase-1 (GPx-1) and decreased expressions of catalase (CAT) heme-oxygenase-1 (HO-1) [75].

### PUFAs Kidney Injury Markers and Structural Alterations in DKD

In DKD, many structural alterations are observed. These include homogenous thickening of the glomerular basement membrane, nodular or diffuse glomerulosclerosis, mesangial cell proliferation and hypertrophy, increased their production of matrix proteins, maladaptive hypertrophy and hyperplasia of the cortical tubular cells, which all ultimately leading to glomerulosclerosis and tubulointerstitial fibrosis [76]. In DKD, PUFA supplementation ameliorated tubulointerstitial fibrosis, extracellular glomerular macrophage infiltration [21], mesangial matrix accumulation [21–23], capillary and tubular dilatation [22], glomerular basal membrane thickening, and podocyte foot process effacement [27]. Additionally decreased kidney tubular injury markers NGAL [26, 34], KIM-1 [26, 32] are associated and reduced by PUFAs.

## Why There Are Divergent Findings?

Although beneficial effects of PUFAs are shown in DKD, these findings are not consistent and the findings are divergent. Indeed, some studies have not shown any effect (neutral). The reasons for these heterogeneous findings are not clear but some speculations can be made. First, the differences in outcome in these previous trials may be attributable to dose, duration, and differences in study design and study subject characteristics. For example, Lee et al. showed that higher intake of omega 3 PUFAs was associated with lower levels of urinary albumin creatinine excretion only in participants with values of Hb1c above the median (7.7%) [38]. Other studies have shown that RAS inhibition is important regarding the impact of PUFAs [34, 40]. Treatment duration is also important. A recent meta-analysis showed that treatment with least 24 weeks is needed to demonstrate reduction in proteinuria and period less than 24 weeks failed to show significant difference in proteinuria [15]. Determination of PUFA intake is important. While most studies use dietary questionnaires [32, 36, 37, 41, 43] other determine compliance with incorporation of PUFAs into phospholipids [18]. The type of PUFAs are also differed between studies. Although both n-3 PUFAs and n-6 PUFAs belong to the same family of fatty acids their mechanisms of actions may differ. It is also important that the Western diet has a high content of n-6 PUFAs, especially linoleic acid (C18:2n-6, LA), and a low content of n-3 PUFAs (particularly EPA and DHA). EPA and DHA as inflammation modulating agents. EPA/DHA relatively decrease arachidonic acid concentration in tissues and compete with as a substrate of the COX-2 and 5-LOX enzymes [77, 78].

These factors may explain the difference between studies.

## Unknowns and Perspectives

The literature regarding PUFAs and DKD contains unknowns. As suggested above, the duration and the type of PUFAs supplementation are not known clearly. The relationship between PUFAs and hard outcomes such as coronary artery disease, myocardial infarction, heart failure, cerebrovascular events are not known. Additionally, the relationship between PUFAs and new antidiabetic drugs (GLP1-RA, SGLT2i, nsMRAs) are not known. Importantly, the side effect profile needs to be clearly established. For example, the large trials have shown that there may be increased risk of atrial fibrillation and bleeding risk with high-dose omega-3 formulations [79, 80]. These are important concerns given that DKD patients carry high baseline cardiovascular and arrhythmogenic burdens.

The reasons for heterogeneity among studies are not known exactly. However, studies used different formulations like EPA ethyl ester (icosapent ethyl), balanced EPA/DHA mixtures (Omacor/Lovaza). Also, the distinct physiological actions of EPA versus DHA likely account for the observed heterogeneity. Lastly, determination methods of intake such as direct measurement or dietary food surveys may be responsible for heterogeneous results.

The studies performed so far opens new areas of research. Firstly, it needs to be determined the impact of PUFAs supplementation specifically on different cell types including glomerular cells, endothelial cells, tubular cells or mesangial cells in the specific context of DKD. Second, whether supplementation of PUFA impacts hard outcomes such as cardiovascular disease, morbidity and mortality should be tested in hard outcomes. Third, the side effect profile of PUFAs supplementation in DKD needs clear documentation. Fourth, the differential impact of Omega 3 and omega 6 fatty acids needs to be studied.

## Conclusions

Some studies regarding PUFAs intake in DKD shows promising results. The beneficial effects of PUFAs were more evident in experimental studies and included decrease of BPs, inflammation, oxidative stress and improvement of structural alterations. However, clinical studies are more heterogeneous and showed inconsistent results. The current guidelines do not routinely recommend use of PUFAs in DKD due to heterogeneous results and unknown issues such as exact duration and type of PUFAs relationship with hard outcomes and other glucose lowering treatments. Future studies are needed to highlight these unknown areas and to explain heterogeneity between studies.

## Key References

- Elbarbary NS, Ismail EAR, Mohamed SA: Omega-3 fatty acids supplementation improves early-stage diabetic nephropathy and subclinical atherosclerosis in pediatric patients with type 1 diabetes: A randomized controlled trial. Clin Nutr 2023, 42(12):2372–2380.
  - ○ This randomized-controlled trial showed that omega-3 supplementation improved glycemic control, dyslipidemia and delayed disease progression and subclinical atherosclerosis in patients with type 1 diabetes.
- Lin SP, Chen CM, Wang KL, Wu KL, Li SC: Association of Dietary Fish and n-3 Unsaturated Fatty Acid Consumption with Diabetic Nephropathy from a District Hospital in Northern Taiwan. Nutrients 2022, 14(10).
  - ○ This study showed that a diet that incorporates high-fat fish, shellfish, soybean products, can mitigate DN risk in patients with Type 2 DM.
- Tian S, Guo T, Qian F, et al.: Fish Oil, Plasma n-3 PUFAs, and Risk of Macro- and Microvascular Complications Among Individuals With Type 2 Diabetes. J Clin Endocrinol Metab 2025, 110(5):e1687-e1696.
  - ○ In this 13.2 years of follow-up study it was demonstrated that habitual use of fish oil supplementation and higher plasma omega 3 polyunsaturated fatty acids levels, were associated with lower risks of macrovascular and microvascular complications among individuals with type 2 diabetes.

## Data Availability

No datasets were generated or analysed during the current study.
